# Palladium-Phosphide-Modified Three-Dimensional Phospho-Doped Graphene Materials for Hydrogen Storage

**DOI:** 10.3390/ma16124219

**Published:** 2023-06-07

**Authors:** Yiwen Chen, Habibullah, Guanghui Xia, Chaonan Jin, Yao Wang, Yigang Yan, Yungui Chen, Xiufang Gong, Yuqiu Lai, Chaoling Wu

**Affiliations:** 1 State Key Laboratory of Clean and Efficient Turbomachinery Power Equipment, Deyang 618000, China; chenyiwen@dongfang.com (Y.C.); laiyuqiu2020@163.com (Y.L.); 2 Dongfang Electric Corporation Dongfang Turbine Co., Ltd., Deyang 618000, China; 3 College of Materials Science and Engineering, Sichuan University, Chengdu 610064, China; habibullah@stu.scu.edu.cn (H.); xiagh1994@163.com (G.X.); jinchaonnan@163.com (C.J.); 4 Engineering Research Center of Alternative Energy Materials & Devices, Ministry of Education, Chengdu 610064, China; wangyao516@scu.edu.cn (Y.W.); yiyang.yan@scu.edu.cn (Y.Y.); chenyungui@scu.edu.cn (Y.C.); 5 Institute of New Energy and Low-Carbon Technology, Sichuan University, Chengdu 610065, China; 6 Technology Innovation Center of Hydrogen Storage-Transportation and Fueling Equipments for State Market Regulation, Chengdu 610100, China

**Keywords:** palladium phosphide, P-doped graphene, hydrogen storage, hydrogen adsorption

## Abstract

The development of efficient hydrogen storage materials is crucial for advancing hydrogen-based energy systems. In this study, we prepared a highly innovative palladium-phosphide-modified P-doped graphene hydrogen storage material with a three-dimensional configuration (3D Pd_3_P_0.95_/P-rGO) using a hydrothermal method followed by calcination. This 3D network hindering the stacking of graphene sheets provided channels for hydrogen diffusion to improve the hydrogen adsorption kinetics. Importantly, the construction of the three-dimensional palladium-phosphide-modified P-doped graphene hydrogen storage material improved the hydrogen absorption kinetics and mass transfer process. Furthermore, while acknowledging the limitations of primitive graphene as a medium in hydrogen storage, this study addressed the need for improved graphene-based materials and highlighted the significance of our research in exploring three-dimensional configurations. The hydrogen absorption rate of the material increased obviously in the first 2 h compared with two-dimensional sheets of Pd_3_P/P-rGO. Meanwhile, the corresponding 3D Pd_3_P_0.95_/P-rGO-500 sample, which was calcinated at 500 °C, achieved the optimal hydrogen storage capacity of 3.79 wt% at 298 K/4 MPa. According to molecular dynamics, the structure was thermodynamically stable, and the calculated adsorption energy of a single H_2_ molecule was −0.59 eV/H_2_, which was in the ideal range of hydrogen ad/desorption. These findings pave the way for the development of efficient hydrogen storage systems and advance the progress of hydrogen-based energy technologies.

## 1. Introduction

Energy is one of the key ingredients in every nation’s economic prosperity. As a consequence of population expansion and evolving lifestyles, global requirements are progressively escalating while encountering environmental problems and an alarming resource depletion rate. The damage that fossil fuel usage has caused to the environment has increased the demand for clean, sustainable energy [1,2,3]. Hydrogen, as a promising new energy source to replace fossil fuels, possesses the advantages of high energy density and zero carbon emissions [4,5]. Hydrogen is expected to be a highly effective alternative fuel with a wide range of applications in the future. The key to applying hydrogen energy in the market is to develop a safe and economical hydrogen storage technology [6]. Compared with gaseous and liquid hydrogen storage, solid hydrogen storage, with its much higher safety, is regarded as the most promising hydrogen storage method [7,8,9]. Among all hydrogen storage materials, carbon materials, especially graphene, have been widely investigated because of their excellent physical and chemical properties [10,11,12,13,14]. Recent studies have also shown the promising photocatalytic activities of various nanocarbon-based materials for hydrogen production. Carbon-based nanomaterials such as graphene, carbon nanotubes, and carbon nitride have been extensively investigated due to their unique properties and high surface area [15,16]. However, pure carbon materials have low reactivity, which is limited to promoting hydrogen dissociation and subsequent chemical adsorption [17,18]. Graphene, when doped with various metal-based elements, has shown promising catalytic properties for hydrogen production. Recent research has focused on investigating different metal dopants to enhance the catalytic activity of graphene. For instance, Wang et al. demonstrated efficient hydrogen evolution using platinum-deposited N-doped graphene, attributing the enhanced activity to the strong metal–support interaction [19]. With the spillover effect of transition group elements such as palladium, the reversible hydrogen storage capacity of carbon materials can be further improved [20,21]. The spillover process primarily involves the dissociation of hydrogen molecules on the catalyst, the migration of hydrogen atoms from the catalyst to the substrate, and the subsequent diffusion of hydrogen atoms on the surface of the substrate. The introduction of heteroatoms in doped nanomaterials has also been studied extensively to understand their impact on the selectivity and yield of hydrogen production. In their review article, Putri et al. highlighted the significant role of heteroatom formation in modulating photocatalytic activities [22]. Additionally, Dobrota et al. explored the effect heteroatoms such as phosphorus atoms doped on the carbon matrix, which could modify the structure of carbon-based materials, thus increasing the adsorption sites of spillover hydrogen atoms and further enhancing the hydrogen storage capacity of carbon materials [23]. In our previous work, a 2D Pd_3_P/P-rGO composite was prepared that showed an improved hydrogen storage capacity compared with pure graphene [24].

However, as a two-dimensional material with a thin sheet structure, graphene sheets tend to stack together spontaneously due to van der Waals forces and π-π bond interactions [25,26]. Irreversible aggregation and accumulation could happen, which are unfavorable for gas diffusion. Thus, their hydrogen storage kinetics and capability may deteriorate seriously. Therefore, hindering the aggregation of graphene sheets is a crucial challenge when realizing the application of graphene as a promising solid hydrogen storage material.

Furthermore, adsorption is also a mass transfer process. Increasing the specific surface area of materials can improve the adsorption sites and subsequent capacity. Constructing a three-dimensional graphene network may provide a valid solution to prevent the graphene sheets from aggregating. The unique three-dimensional lamellar folds and pore structure of graphene can effectively prevent this interlayer accumulation, which is the intrinsic reason for the stable existence of 3D graphene [27]. Bi et al. constructed a three-dimensional B-doped graphene framework decorated with Li ions. It possessed an excellent hydrogen storage capacity of about 12.9 wt%, calculated using DFT (density function theory) [28]. Tylianakis et al. designed a novel 3D nanostructured porous nanotube material with a high hydrogen storage capacity of 20 wt% at 77 K by a multiscale theoretical approach [29]. In addition, three-dimensional graphene with multilevel porous structures can also effectively promote mass transfer [30]. Yan et al. researched a 3D rGO-Mn_3_O_4_ nanosheet hybrid decorated with Pd for hydrogen evolution. They confirmed that the 3D network architecture was favorable for the mass transport of produced hydrogen [31]. Jiang et al. designed a new theoretical type of 3D graphene bubble structure for hydrogen storage, and the molecular dynamics of hydrogen storage were enhanced because of the 3D structure [32]. Thus, since 3D graphene has an excellent porous structure, during hydrogen absorption/desorption, the movement of hydrogen molecules to/from the active centers could become easier in 3D graphene, which would result in accelerated kinetics for hydrogen storage compared with 2D graphene [32].

Based on our previous work, herein, a 3D Pd/P co-modified graphene hydrogel material was successfully prepared by a hydrothermal method, and 3D Pd_3_P_0.95_/P-rGO was obtained by thermal reduction in a H_2_/Ar atmosphere. Palladium phosphide was grown directly on the three-dimensional graphene substrate. This 3D structure could effectively promote mass transfer. The multi-stage gap structure helped the gas be transported to/separate from the reaction center to promote molecular dynamics. Therefore, the as-prepared 3D Pd_3_P_0.95_/P-rGO-500, which was calcinated at 500 °C, achieved a hydrogen storage capacity of 3.79 wt% at 298 K/4 MPa. Meanwhile, the hydrogen absorption rate of the material increased obviously in the first 2 h compared with 2D sheets of Pd_3_P/P-rGO.

## 2. Material Preparation

### 2.1. Preparation of GO Solution

The modified Hummers method was used to produce graphene oxide. Briefly, 5 g of graphite powders and 1 g of NaNO_3_ were first added to a 1000 mL beaker. After that, 100 mL of concentrated sulfuric acid was slowly added to the beaker, and 12 g of KMnO_4_ was slowly incorporated with continuous high-speed stirring. The as-obtained mixture was thoroughly stirred in an ice water bath for 8 h. Afterward, 3 vol% H_2_O_2_ solution was added to the beaker to completely reduce the excess KMnO_4_ until the brown solution turned yellow. The GO solution after the reaction was kept static at room temperature and was observed to change from bright yellow to brown with obvious stratification. The supernatant was poured out, and the precipitate was washed twice with concentrated hydrochloric acid. Then, deionized water was added repeatedly for cleaning until the pH value of the solution was close to neutral. The GO solution with 3 wt% content was obtained after filling to a constant volume of 1000 mL.

### 2.2. Preparation of 3D-Configuration Pd_3_P_0.95_/P-rGO at Different Thermal Reduction Temperatures

We mixed 33 mL GO solution, 78 mg Pd(OAc)_2_, 120 μL phytic acid, and 33 mL deionized water in a 100 mL beaker using ultrasound for 30 min. Then, the above solution was transferred to a 100 mL Teflon lining. After that, the lining was sealed in a stainless-steel high-pressure reaction kettle for 12 h for the hydrothermal reaction, and the temperature was kept at 160 °C. After the reaction, the cylindrical hydrogel material was freeze-dried and then heated to 900 °C, 700 °C, 500 °C, and 300 °C for 1 h in a 10 vol% H_2_/Ar atmosphere. The 3D-configuration Pd_3_P_0.95_/P-rGO prepared at different thermal reduction temperatures is denoted as 3D Pd_3_P_0.95_/P-rGO-Y (Y = 300, 500, 700, 900), where the numbers 300, 500, 700, and 900 represent the reduction temperatures.

### 2.3. Computational Methodology

A graphene surface model consisting of 60 atoms was utilized in this study, and a vacuum layer of 15 Å was added along the Z-direction to avoid any interactions between neighboring layers. The single graphene model with 59 atoms was obtained by substituting a carbon atom with a phosphorus atom from the graphene sheet and attaching the oxygen atom to the slab, thus creating phosphorus-reduced graphene oxide (P-rGO). The structure was subsequently optimized. The study employed density functional theory (DFT) to calculate the binding energy, H_2_ adsorption energy, transition state, and energy barrier, with a focus on characterizing the hydrogen migration through the Pd_3_P cluster sites to the P-rGO surface. The Vienna Ab initio Simulation Package (VASP) was used to perform the calculations. Ab initio molecular dynamics (AIMD) was utilized to study the model. The exchange and correlation interactions were described using generalized gradient approximation (GGA) in the form of the Perdew–Burke–Ernzerhof (PBE) functional. The calculations used a Brillouin region of 5 × 5 × 1 as the k-point value; the geometry optimization structure was obtained by relaxation until the force on each atom was less than 0.02 eV/Å; and the convergence criterion for energy was set as 1 × 10^−4^ eV. The cutoff energy was 450 eV, and all calculations considered spin polarization. The study employed an equation to determine the adsorption energy of the H_2_ molecule, taking into account the vibration effect for a gas phase, only as it is nearly negligible for a solid system.

$$E_{\left(ads\right)}=\left(E_{\left(H2+Cluster+substarte\right)}+△ZPE\right)-E_{\left(cluster+substrate\right)}-\left(E_{\left(H2\right)}+△ZPE\right)$$

where $E_{\left(ads\right)}$ represents the H_2_ adsorption energy, $E_{\left(H2+cluster+substarte\right)}$ represents the total energy of a system, $E_{\left(cluster+substarte\right)}$ is the total energy of the cluster decorated substrate, $E_{\left(H2\right)}$ is the total energy of a single H_2_ molecule, and △ZPE is the zero-point energy correction.

## 3. Results and Discussion

**Figure 1:**
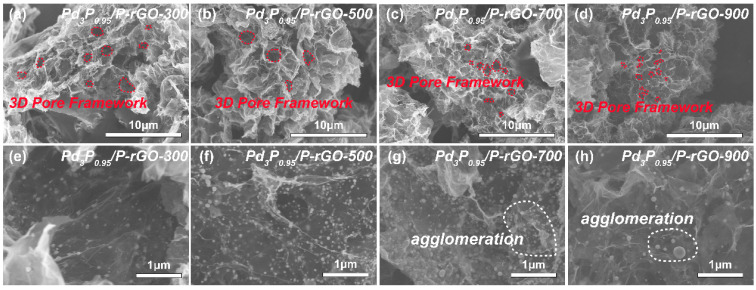
SEM images of 3D Pd_3_P_0.95_/P-rGO prepared at different thermal reduction temperatures (300, 500, 700, 900 °C): (**a**,**e**) Pd_3_P_0.95_/P-rGO-300, (**b**,**f**) Pd_3_P_0.95_/P-rGO-500, (**c**,**g**) Pd_3_P_0.95_/P-rGO-700, and (**d**,**h**) Pd_3_P_0.95_/P-rGO-900.

It can be seen from the model diagram that the configuration was an accumulation of 2D graphene sheets with different shapes, as revealed in Figure S1a,b. In Figure S1c,d, the optical images display the morphology of the 3D Pd_3_P_0.95_/P-rGO, mainly showing a fluffy columnar structure. The SEM images of the as-prepared materials (Figure 1) show that there were obvious large particles in the Pd_3_P_0.95_/P-rGO-900 and Pd_3_P_0.95_/P-rGO-700 samples, which may have been caused by high-temperature agglomeration. It has previously been reported that increasing the thermal reduction temperature leads to the formation of larger particles [33]. Compared with the former two samples, the nanoparticles in the Pd_3_P_0.95_/P-rGO-500 sample were significantly finer and more evenly distributed, which indicated that reducing the thermal reduction temperature could hinder the growth of the particles and agglomeration in the material. When the thermal reduction temperature dropped to 300 °C, Pd_3_P_0.95_ with an obvious hydrogen spillover capacity could not be obtained, which was not conducive to improving the hydrogen storage performance of the material. Therefore, 500 °C was selected as the final temperature for thermal reduction in the subsequent experiment. According to our previous article, in the synthesis of Pd_3_P-decorated P-doped materials, the loading of palladium phosphide particles on the surface was predicted using a field emission scanning electron microscope [24].

**Figure 2:**
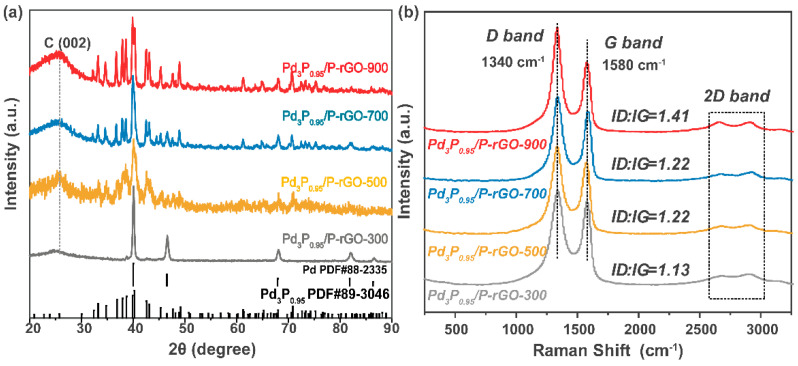
(**a**) XRD patterns and (**b**) Raman spectra of 3D Pd_3_P_0.95_/P-rGO prepared at different thermal reduction temperatures (300, 500, 700, 900 °C).

Figure 2a shows the XRD patterns of Pd_3_P_0.95_-modified P-doped graphene prepared at 900 °C, 700 °C, 500 °C, and 300 °C. Through a comparison with the standard PDF card, we found that after adjusting the amount of P, the Pd_3_P_0.95_ crystal phase was present in the Pd_3_P_0.95_/P-rGO-900 sample. When the thermal reduction temperature decreased, the crystal phase gradually changed into mixed phases of Pd and Pd_3_P_0.95_. However, the Pd content was trivial, and the main phase was still Pd_3_P_0.95_. The thermal reduction temperature had an obvious influence on the grain size, which was calculated by the Scherrer formula, and the average grain sizes were as follows: 29.8 nm at 900 °C > 27 nm at 700 °C > 25.7 nm at 500 °C > 23.5 nm at 300 °C. The characterization of the material’s crystal structure can be found in our previous TEM analysis [24], as the synthesis conditions were similar in both studies; the TEM analysis revealed the crystallinity. Jiang et al. also reported the influence of the thermal reduction temperature on the crystallite size; as the thermal reduction temperature of the Ni_2_P/Na-M41 samples increased from 450 to 600 °C, the crystallite size also increased [33].

Figure 2b shows the Raman spectra of the 3D-configuration Pd_3_P_0.95_/P-rGO-Y (Y = 300, 500, 700, 900 °C) prepared at different thermal reduction temperatures. With the increase in the thermal reduction temperature, more oxygen-containing groups in the graphene were reduced, thus forming defects similar to vacancies. At the same time, the doping degree of P atoms further increased, leading to an increment in the I_D_/I_G_ value of the sample (see Figure S4), indicating that the defect degree was greatly improved, favoring hydrogen occupancy.

**Figure 3:**
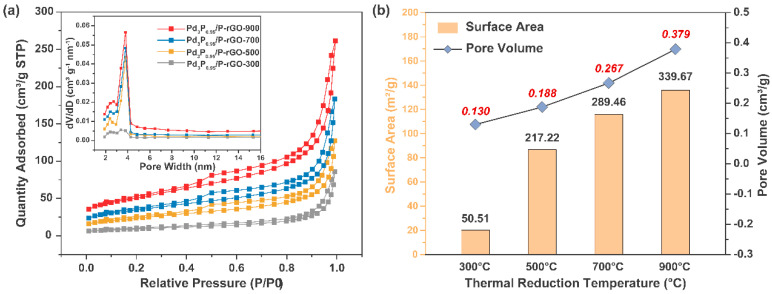
(**a**) N_2_ adsorption–desorption curves (inset shows pore size distribution) and (**b**) summary of specific surface area and pore volume of 3D Pd_3_P_0.95_/P-rGO-Y (Y = 300, 500, 700, 900 °C) prepared at different thermal reduction temperatures.

Figure 3 exhibits the N_2_ adsorption–desorption curves of the 3D-configuration Pd_3_P_0.95_/P-rGO-Y (Y = 300, 500, 700, 900) prepared at different thermal reduction temperatures, and the inset shows the particle size distribution. The N_2_ adsorption–desorption curves of the Pd_3_P_0.95_/P-rGO-Y (Y = 300, 500, 700, 900) at 77 K showed the characteristics of a type IV isothermal adsorption line, and an obvious hysteresis phenomenon occurred during the N_2_ desorption of the four samples, which indicated that the material had a rich pore structure, that is, a large number of mesopores existed. We found that the average pore sizes of the four samples were all distributed around ~3.8 nm, except for Pd_3_P_0.95_/P-rGO-300, in which the number of 3.8 nm mesopores was lower, as shown in the inset in Figure 3a. Figure 3b shows the specific surface area and pore volume of Pd_3_P_0.95_/P-rGO-Y (Y = 300, 500, 700, 900). Pd_3_P_0.95_/P-rGO-900 had the largest surface area and pore volume at 339.67 m^2^/g and 0.379 cm^3^/g, respectively. The specific surface area and pore volume of Pd_3_P_0.95_/P-rGO-300 decreased to 50.51 m^2^/g and 0.130 cm^3^/g, respectively, when the thermal reduction temperature dropped to 300 °C. With the decrease in the thermal reduction temperature, Pd ions could not be reduced to obtain the expected palladium phosphide, and nor could the oxygen-containing groups be completely reduced.

Combined with the Raman test results in Figure 2b, the defects in the Pd_3_P_0.95_/P-rGO-300 were reduced, leading to an obvious reduction in the specific surface area and pore volume. This showed that both the defect degree and the micro-structure in the material were closely related to the thermal reduction temperature. The influence of metal and non-metal doping on the surface area and pore characteristics of particles was investigated by Safia et al. [34]. In a separate study, Vieira and colleagues [35] demonstrated that the addition of 0.5 wt% Ce and 0.15 wt% Nd resulted in a significant enhancement of over 100% in the S_BET_ of the catalysts. Furthermore, Sahoo et al. [36] reported an increase in both the surface area and volume porosity following the introduction of dopants into the catalyst. Ting et al. highlighted in their publication that the catalyst was profoundly influenced by a reduction in temperature, consequently impacting its catalytic activity. The authors found that excessively low reduction temperatures, such as 350 °C, led to a significant decrease in catalytic efficiency. However, when the catalysts were calcined at 450 °C and subsequently reduced at 600 °C, they exhibited remarkable catalytic activity, achieving an efficiency of up to 74.76% [37].

**Figure 4:**
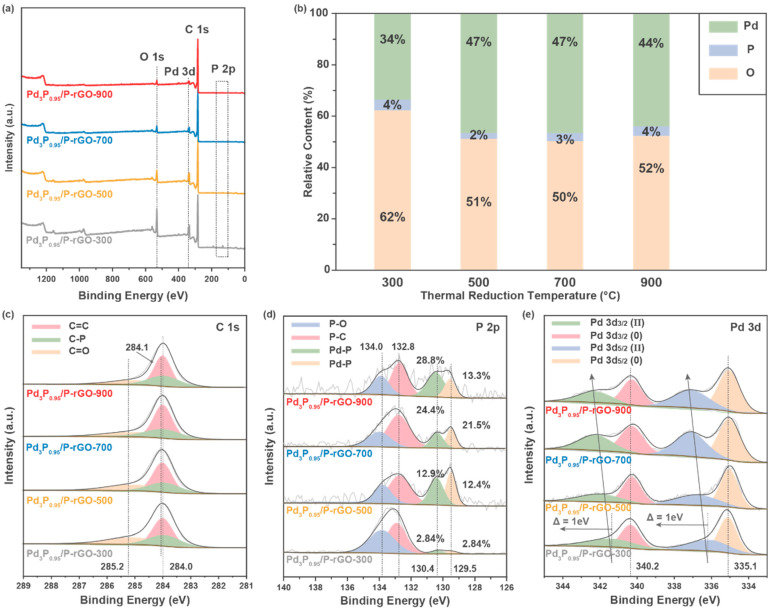
XPS spectra of 3D Pd_3_P_0.95_/P-rGO-Y (Y = 300, 500, 700, 900 °C) prepared at different thermal reduction temperatures: (**a**) survey spectra, (**b**) the relative content of each element, (**c**) C 1s, (**d**) P 2p, and (**e**) Pd 3d orbitals.

To further study the electronic structure and chemical valence of the elements on the surface of the material, X-ray photoelectron spectroscopy (XPS) was performed on the 3D-configuration Pd_3_P_0.95_/P-rGO-Y (Y = 300, 500, 700, 900) samples, as shown in Figure 4. Figure 4a shows the total XPS spectrum, in which the peaks corresponding to the P 2p (~130 eV), C 1s (~280 eV), Pd 3d (~330 eV), and O 1s (~530 eV) orbitals can be observed, indicating that these elements were present on the surface of the sample. Figure 4b exhibits the relative content of each element on the sample surface. With the increase in the thermal reduction temperature, the relative content of O in the material decreased, indicating that more oxygen-containing groups in the GO were reduced. At the same time, the relative content of Pd increased, which indicated that a higher thermal reduction temperature could make the Pd more fully reduced and phosphated. In the C 1s spectrum displayed in Figure 4c, the peaks at 284.0 eV and 285.2 eV correspond to the C=C bond and C=O bond, respectively, which are characteristic peaks of rGO, while the peak located at 284.1 eV corresponds to the C-P peak, indicating that P was successfully doped into the rGO. In the P 2p spectrum, the peaks at 132.5 eV and 133.5 eV correspond to P-C and P-O bonds, respectively, which indicate P doping in the graphene [38]. The peaks at 129.4 eV and 130.3 eV came from P 2p_3/2_ and P 2p_1/2_, respectively, which corresponded to Pd-P bonds, indicating the successful synthesis of Pd_3_P_0.95_ particles in the material [39]. The EDX analysis also confirmed the presence of C, O, Pd, and P in the sample; see Figure S5 for elemental analysis. The Pd 3d spectrum is exhibited in Figure 4e, and the peaks located at 335.1 eV and 340.2 eV belong to the 3d_5/2_ and 3d_3/2_ orbits of Pd^(0)^, respectively. The peaks located near 336 eV and ~342 eV correspond to the 3d_5/2_ and 3d_3/2_ orbitals of Pd^(II)^, indicating that Pd with a high valence state existed due to the formation of a Pd-O bond [40].

**Figure 5:**
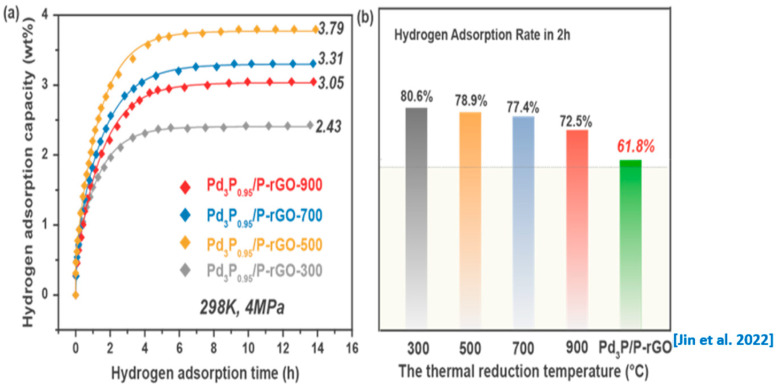
(**a**) Hydrogen adsorption kinetic curves and (**b**) hydrogen adsorption rate in the first 2 h of 3D Pd_3_P_0.95_/P-rGO-Y (Y = 300, 500, 700, 900 °C) and 2D Pd_3_P/P-rGO from [24] at 4 MPa/298 K.

Figure 5a,b show the hydrogen absorption kinetic curves of the 3D-configuration Pd_3_P_0.95_/P-rGO-Y (Y = 300, 500, 700, 900) prepared at different thermal reduction temperatures under the conditions of 4 MPa/298 K and the corresponding hydrogen storage capacity. With the decrease in the thermal reduction temperature, the hydrogen storage capacity of the material increased first and then decreased, reaching the maximum value (3.79 wt%) when the thermal reduction temperature was 500 °C. This was because reducing the thermal reduction temperature could effectively prevent the nanoparticles in the material from agglomerating and subsequently falling off. Thus, the size of the modified nanoparticles in the material was well controlled, and the hydrogen spillover capacity was enhanced, contributing to an obvious increase in the hydrogen storage capacity. However, we found that the Pd could not be fully phosphatized at 300 °C. Therefore, the hydrogen spillover effect of the Pd_3_P_0.95_/P-rGO-300 was greatly reduced, and the hydrogen storage performance decreased sharply.

Figure 5b shows the percentage hydrogen storage capacity in the first 2 h during the hydrogen absorption process over the total hydrogen storage capacity, to compare the hydrogen absorption kinetics of the materials. We found that the hydrogen absorption rate decreased with the increase in the thermal reduction temperature, and Pd_3_P_0.95_/P-rGO-900 showed the lowest hydrogen absorption amount of 72.5% in the first 2 h, indicating that the hydrogen absorption rate was directly related to the particle size of the modified particles in the material. The smaller the particle size, the higher the hydrogen absorption rate. The hydrogen absorption rates of the four samples were significantly higher than in our previous work for 2D Pd_3_P/P-rGO (61.8%) [24]. The improvement in the hydrogen absorption kinetics of Pd_3_P_0.95_/P-rGO was mainly due to the formation of a 3D structure, which could greatly enrich the pore structure in the material and provide channels for hydrogen diffusion, thus accelerating the hydrogen absorption process. During the spillover process, the hydrogen molecules underwent dissociation on the Pd_3_P_0.95_ nanoparticles and subsequently migrated to the surface of the graphene. The dissociated hydrogen species, chemisorbed onto the surface, involved covalent interactions, whereas the absorption of hydrogen molecules onto the graphene was governed by weaker van der Waals forces, which represented non-covalent interactions. Additionally, it is noteworthy that the environmental temperature and pressure exerted a significant influence on the hydrogen storage capacity. Experimental results indicate that reducing the test temperature and increasing the pressure of hydrogen has a favorable effect on hydrogen adsorption [41].

The binding energies of Pd_3_P, P-rGO, and P-doped graphene (P-G) were calculated based on DFT, and the optimized structures are shown in Figure S5a,b. The calculated binding energies were −1.71 between Pd_3_P and P-rGO and −2.14 eV between Pd_3_P and P-G. The results showed that the reduction of the oxygen atoms could increase the binding energy of the cluster and slab. The higher the binding energy, the lower the chance of the agglomeration of transition metal (TM) atoms and vice versa. DFT simulations were performed at 0 K, and the structural integrity of the system had to be checked at room temperature (300 K). In our case, wherein Pd_3_P was attached to the P-rGO, it was critical that the TM atoms did not dislodge from the P-rGO at higher temperatures. The AIMD simulations considered these concerns. The simulations were carried out at a temperature of 300 K in a canonical ensemble (NVT) for 1 ps with a time step of 1 fs. The MD snapshots of Pd_3_P/P-rGO are shown in Figure S6, indicating that the material was quite stable. Even at 300 K, the movement of the Pd_3_P cluster and the change in the C-C bond length were negligible, highlighting the thermal stability of the structure and supporting the material’s practical application in hydrogen storage systems. Because the probability of metal atoms or clusters aggregating on supports is a major downside of transition metals in graphene-based materials, we know that the metal–metal interactions are stronger than chemical interactions between metal atoms and carbon atoms in the support [42]. This MD investigation demonstrated the material’s thermal stability, which showed its ability to store hydrogen. Additionally, in unstable materials, metal–hydrogen complex desorption often competes with H_2_ desorption [43]. In our previous study, it was observed that the hydrogen storage capacity of the material decreased from 3.66 wt% to 2.63 wt% at a temperature of 298 K and a pressure of 4 MPa after undergoing four cycles of hydrogen adsorption. This reduction in capacity could be attributed to the formation of metal hydrides within the material [24].

**Figure 6:**
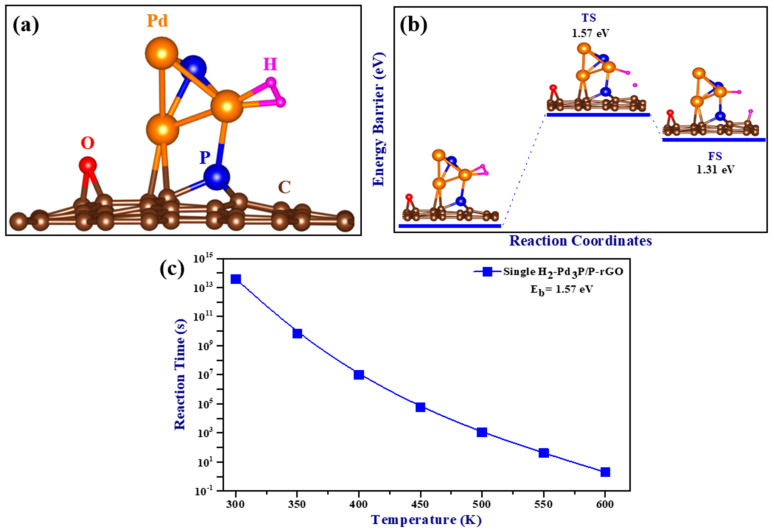
(**a**) The optimized structure of H_2_ adsorbed on Pd_3_P/P-rGO; (**b**) the minimum energy path (MEP) for the spillover of hydrogen on Pd_3_P/P-rGO; and (**c**) single H_2_-Pd_3_P/P-rGO spillover reaction time as a function of temperature (TS: transition state, FS: final state).

The optimized structure of a single adsorbed H_2_ molecule is shown in Figure 6a, and the calculated adsorption energy was −0.59 eV/H_2_ when the zero-point energy correction was taken into account. A negative adsorption energy value indicates the attraction of H_2_ molecules to the cluster, implying favorable adsorption. Adsorption mechanisms that fall between physical and chemical adsorption are required for the successful implementation of hydrogen storage in ambient conditions. Previous research has suggested an adsorption energy range of −0.20 to −0.60 eV [44], which agrees with our calculated adsorption energy. This system’s adsorption energy range corresponded to an intermediate physisorbed–chemisorbed state, which was advantageous for working under ambient conditions. Interestingly, the observed H-H bond length was 0.85 Å, longer than that of the free hydrogen molecule (0.75 Å), and the bond length between Pd and H ranged from 1.76 to 1.77 Å. However, when compared to an isolated H_2_ molecule, the stretching of the H-H bond length was found to be minimal, with a difference of less than 0.3 Å [45], indicating that the molecular nature of H_2_ was preserved during adsorption and the association between the H_2_ molecule and the Pd atom was of the Kubas type.

A crucial stage in hydrogen storage is the hydrogen spillover mechanism, which involves the dissociation of H_2_ molecules and the migration of H atoms from the site of dissociation to the supporting substrate. In this study, the spillover process of a single hydrogen molecule adsorbed on Pd_3_P/P-rGO was computed using the nudged elastic band (NEB) and climbing image nudged elastic band (CINEB) [46] methods. The minimum energy path is plotted as a function of the reaction coordinates in Figure 6b. With a total reaction energy of −1.31 eV and a calculated migration barrier of 1.57 eV for a single H_2_ adsorbed on Pd_3_P/P-rGO, spillover occurred at higher temperatures due to the higher energy migration barrier. The DFT results were in line with experimental data showing that Pd_3_P_0.95_/P-rGO-300’s hydrogen spillover effect was substantially reduced. The relationship between temperature and spillover reaction time is depicted in Figure 6c. The migration time decreased as the temperature increased, and the relationship between the temperature and the energy barrier was inverse. The hydrogen storage capacities of the 3D Pd_3_P_0.95_/P-rGO-Y (Y = 300, 500, 700, 900 °C) samples were much higher than those of some graphene-based materials previously reported in the literature (see Table 1) under ambient conditions. Furthermore, the scarcity of research on 3D graphene-based materials for hydrogen storage underscores the novelty of this work. Notably, there is a lack of literature reporting the absorption kinetics and mass transfer properties of 3D configurations.

## 4. Conclusions

In conclusion, this study presented a highly innovative approach to the development of hydrogen storage materials by preparing a three-dimensional configuration of palladium-phosphide-modified P-doped graphene (3D Pd_3_P_0.95_/P-rGO) using a hydrothermal method followed by calcination. The three-dimensional network structure of the material hindered the stacking of graphene sheets and provided channels for improved hydrogen diffusion, thereby enhancing the hydrogen adsorption kinetics and mass transfer process. The research findings demonstrated that the constructed three-dimensional palladium-phosphide-modified P-doped graphene material exhibited significantly higher hydrogen absorption rates in the initial 2 h compared to its two-dimensional counterparts, highlighting the advantages of the three-dimensional configuration. Specifically, the 3D Pd_3_P_0.95_/P-rGO-500 sample, calcinated at 500 °C, achieved an optimal hydrogen storage capacity of 3.79 wt% at 298 K/4 MPa. The thermodynamic stability of the structure was confirmed by molecular dynamics simulations, which also revealed an adsorption energy of −0.59 eV/H_2_ for a single H_2_ molecule, falling within the ideal range for efficient hydrogen adsorption/desorption. The significance of this research lies in its addressing the need for improved graphene-based hydrogen storage to overcome the limitations of pure graphene in hydrogen storage. By exploring three-dimensional configurations, this study opened up new possibilities for the development of efficient hydrogen storage systems and advanced the progress of hydrogen-based energy technologies. The innovative approach presented in this study has the potential to contribute significantly to the advancement of hydrogen-based energy systems, offering a promising avenue for further research and development in this field.

## Figures and Tables

**Table 1:** Comparison of hydrogen storage capacities of 3D Pd_3_P_0.95_/P-rGO-Y (Y = 300, 500, 700, 900 °C) presented in the present work with those of previously reported graphene-based materials.

Materials	Temperature (K)	Pressure ^a^	H_2_ Storage (wt%)	Ref.
3D graphene	77	1 bar	1.4	[47]
Pd-graphene/carbon	298	8 MPa	0.82	[48]
Pd/N-rGO	298	4 MPa	2.90	[49]
Ni/Pd-rGO	293.15	800 mmHg	0.13	[50]
3DHPG-Ni-7.5 nanocomposite	77	5 bar	4.22	[51]
3DHPG-Ni-7.5 nanocomposite	298	5 bar	1.95	[51]
3D Pd_3_P_0.95_/P-rGO-300	298	4 MPa	2.43	This work
3D Pd_3_P_0.95_/P-rGO-500	298	4 MPa	3.79	This work
3D Pd_3_P_0.95_/P-rGO-700	298	4 MPa	3.31	This work
3D Pd_3_P_0.95_/P-rGO-900	298	4 MPa	3.05	This work

^a^ 1 bar = 0.1 MPa, 1 bar = 750.1 mmHg.

## Data Availability

No new data were created or analyzed in this study. Data sharing is not applicable to this article.

## Supplementary Materials

The following supporting information can be downloaded at: https://www.mdpi.com/article/10.3390/ma16124219/s1.
