# Supplementary material for: Palladium-Phosphide-Modified Three-Dimensional Phospho-Doped Graphene Materials for Hydrogen Storage
Source: Materials (Basel). 2023 Jun 7;16(12):4219. doi: 10.3390/ma16124219 (PMC10305715; doi:10.3390/ma16124219)
Supplement: Supplementary file 1 [file materials-16-04219-s001.zip › materials-2415091-supplementary.pdf]

Supplementary information

Palladium phosphide modified three-dimensional phospho-doped graphene materials for hydrogen storage

Yiwen Chen [1,2,†], Habibullah [3,†], Guanghui Xia [3], Chaonan Jin [3], Yao Wang [4,5], Yigang Yan [4,5,6], Yungui Chen [4,5,6], Xiufang Gong [1,2,*], Yuqiu Lai [1,2] and Chaoling Wu [3,4,6,*]

1 State Key Laboratory of Clean and Efficient Turbomachinery Power Equipment, Deyang 618000, China; chenyiwen@dongfang.com (Y.C.); laiyuqiu2020@163.com (Y.L.)

2 Dongfang Electric Corporation Dongfang Turbine Co., Ltd., Deyang 618000, China

3 College of Materials Science and Engineering, Sichuan University, Chengdu 610064, China; habibullah@stu.scu.edu.cn (H.); xiagh1994@163.com (G.X.); jinchaonnan@163.com (C.J.)

4 Engineering Research Center of Alternative Energy Materials & Devices, Ministry of Education, Chengdu 610064, China; wangyao516@scu.edu.cn (Y.W.); yiyang.yan@scu.edu.cn (Y.Y.); chenyungui@scu.edu.cn (Y.C.)

5 Institute of New Energy and Low-Carbon Technology, Sichuan University, Chengdu 610065, China

6 Technology Innovation Center of Hydrogen Storage-Transportation and Fueling Equipments for State Market Regulation, Chengdu 610100, China

* Correspondence: gongxiufang@dongfang.com (X.G.); wuchaoling@scu.edu.cn (C.W.)

† These authors contributed equally to this work.

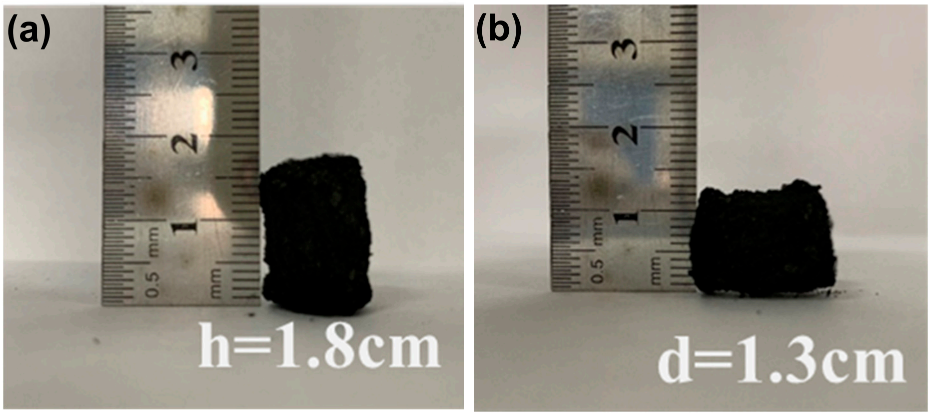

Figure S1: (a-b) Optical images of 3D Pd_3P/P-rGO-Y. (Y=300, 500, 700, 900)

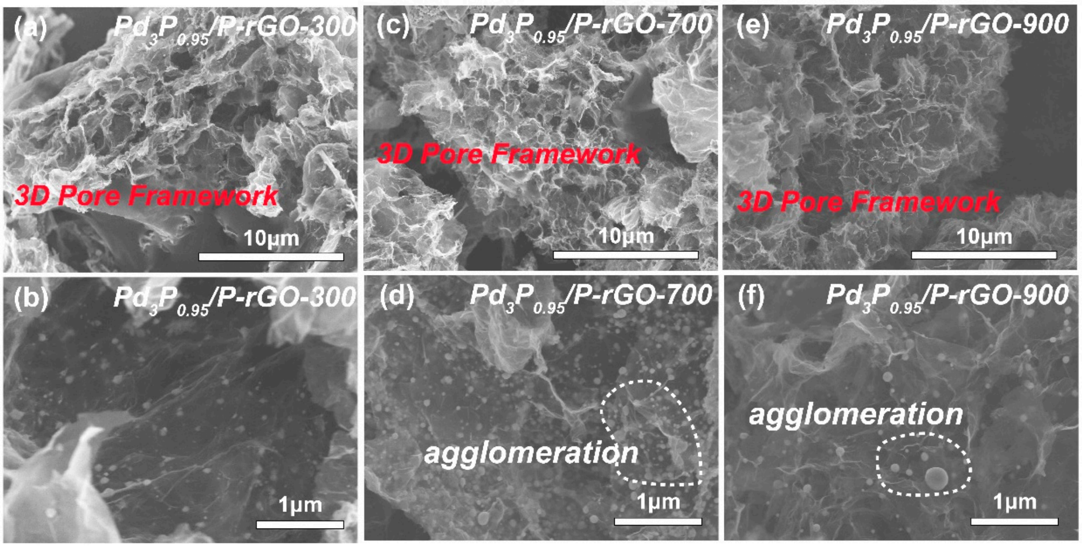

Figure S2: SEM images of 3D $Pd_3P_{0.95}$/P-rGO prepared at different thermal reduction temperatures: (a-b) $Pd_3P_{0.95}$/P-rGO-300, (c-d) $Pd_3P_{0.95}$/P-rGO-700, (e-f) $Pd_3P_{0.95}$/P-rGO-900.

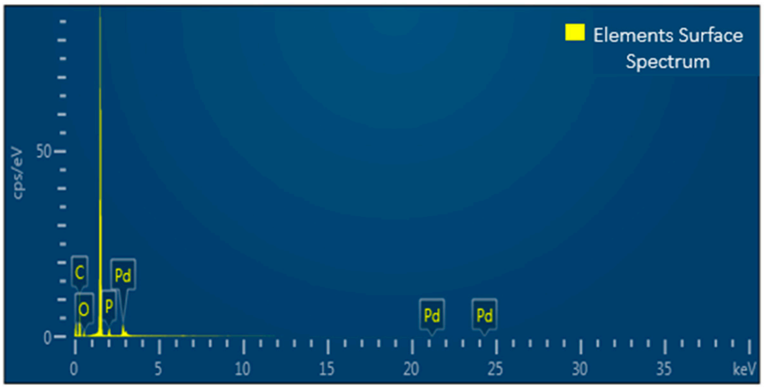

Figure S3: Energy Dispersive X-Ray Analysis (EDX) of the sample to confirm successful doping of P in the $Pd_3P_{0.95}$/P-rGO

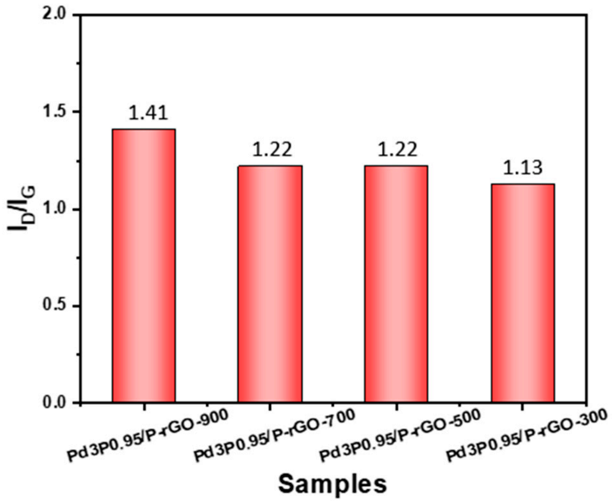

Figure S4: Statistical chart of I_D/I_G values.

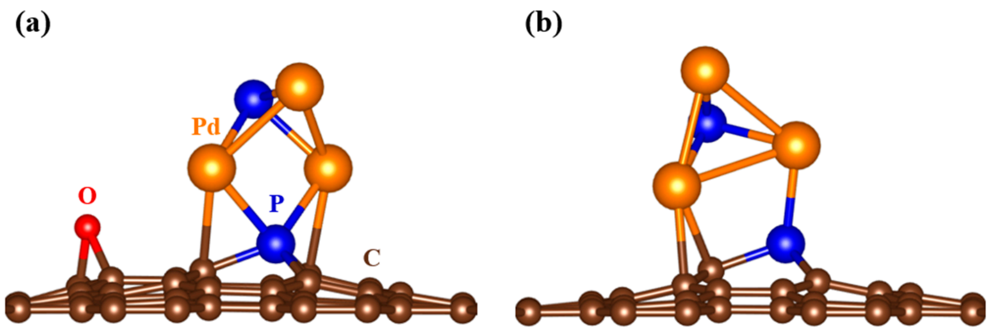

Figure S5: Optimized structure of (a) Pd_3P/P-rGO and (b) Pd_3P/P-G.

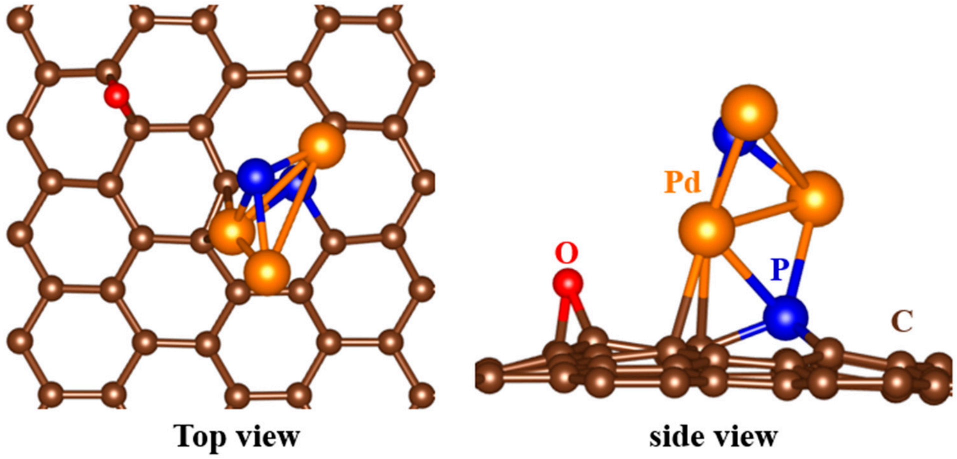

Figure S6: Molecular Dynamics snapshot of Pd_3P/P-rGO at 300K after 1 ps. The structure appears to be stable and there are minimal changes in C-C bond length.
